# Optimization of β-Glucosidase, β-Xylosidase and Xylanase Production by *Colletotrichum graminicola* under Solid-State Fermentation and Application in Raw Sugarcane Trash Saccharification

**DOI:** 10.3390/ijms14022875

**Published:** 2013-01-30

**Authors:** Ana L. R. L. Zimbardi, Cesar Sehn, Luana P. Meleiro, Flavio H. M. Souza, Douglas C. Masui, Monica S. F. Nozawa, Luis H. S. Guimarães, João A. Jorge, Rosa P. M. Furriel

**Affiliations:** 1 Department of Chemistry, Faculty of Philosophy, Sciences and Languages of Ribeirão Preto, University of SãoPaulo, Bandeirantes Avenue, 3900, Ribeirão Preto, SP 14040-901, Brazil; E-Mails: luccilatorre@usp.br (A.L.R.L.Z.); cesar.sehn@hotmail.com (C.S.); luanapm@aluno.ffclrp.usp.br (L.P.M.); flaviosouza@usp.br (F.H.M.S.); 2 Department of Biology, Faculty of Philosophy, Sciences and Languages of Ribeirão Preto, University of São Paulo, Bandeirantes Avenue, 3900, Ribeirão Preto, SP 14040-901, Brazil; E-Mails: dcmasui@usp.br (D.C.M.); lhguimaraes@ffclrp.usp.br (L.H.S.G.); joajorge@ffclrp.usp.br (J.A.J.); 3 Laboratory of Gene Expression and Microbiology, Department of Graduation, University Nilton Lins, Prof. Nilton Lins Avenue, 3259, Manaus, AM 69058-040, Brazil; E-Mail: profmonica.nozawa@gmail.com

**Keywords:** β-glucosidase, β-xylosidase, xylanase, *Colletotrichum graminicola*, sugarcane trash hydrolysis

## Abstract

Efficient, low-cost enzymatic hydrolysis of lignocellulosic residues is essential for cost-effective production of bioethanol. The production of β-glucosidase, β-xylosidase and xylanase by *Colletotrichum graminicola* was optimized using Response Surface Methodology (RSM). Maximal production occurred in wheat bran. Sugarcane trash, peanut hulls and corncob enhanced β-glucosidase, β-xylosidase and xylanase production, respectively. Maximal levels after optimization reached 159.3 ± 12.7 U g^−1^, 128.1 ± 6.4 U g^−1^ and 378.1 ± 23.3 U g^−1^, respectively, but the enzymes were produced simultaneously at good levels under culture conditions optimized for each one of them. Optima of pH and temperature were 5.0 and 65 °C for the three enzymes, which maintained full activity for 72 h at 50 °C and for 120 min at 60 °C (β-glucosidase) or 65 °C (β-xylosidase and xylanase). Mixed with *Trichoderma reesei* cellulases, *C. graminicola* crude extract hydrolyzed raw sugarcane trash with glucose yield of 33.1% after 48 h, demonstrating good potential to compose efficient cocktails for lignocellulosic materials hydrolysis.

## 1. Introduction

Recent years have seen an ever-increasing interest in cellulose and hemicellulose hydrolysis, in an effort to develop a cost-effective process to use lignocellulosic residues as raw materials for the production of value added products, mainly bioethanol [1–3]. In Brazil, a great producer of bioethanol from sugarcane by conventional processes, sugarcane bagasse and trash (cane leaves and tops) appear as attractive options for cellulosic ethanol production. Besides their low cost, these residues are produced in huge amounts at or near the mills, thus reducing transport and infrastructural costs for further processing [4,5]. Usually, most sugarcane bagasse is burned to produce steam to run the plant, and for bioelectricity generation [6]. In contrast, sugarcane trash (SCT), estimated as 140 kg (dry) per sugarcane ton processed, is left in the field after mechanical sugarcane harvesting [4,5]. Thus, although the maintenance of 40%–50% SCT in the field is recommended for soil conservation and disease control [7], the surplus is now considered a promising source for second-generation ethanol production.

The enzymatic treatment is considered the most promising method for an efficient hydrolysis of cellulose in lignocellulosic residues [3,8–10]. However, the high cost of cellulolytic enzymes [1,8,10,11] remains a major bottleneck for cellulosic ethanol production. Moreover, due to the recalcitrance of the lignocellulosic matrix to enzymatic hydrolysis [1,9], costly physicochemical pre-treatments are often necessary [1–3,12].

During the last few years, researchers’ interest has been mostly focused on cellulases and cellulase-based cocktails for cellulose hydrolysis. However, it is now well known that enzymatic cocktails containing both cellulases and hemicellulases are required for an efficient saccharification of cellulose in lignocellulosic residues, resulting in higher yields of fermentable sugars with lower enzyme loads [8,10,13,14]. Xylan is the main hemicellulose in agricultural residues, and its removal from the outer surface of the cellulosic fibers by xylanases evidently improves the accessibility of the cellulose chains to cellulases. The more efficient hydrolysis of cellulose then leads to an enhanced exposure of the xylan chains entrapped between cellulosic fibers to the action of xylanases [10]. Additionally, some studies have also shown that xylan and xyloligosaccharides are potent inhibitors of cellulases [13]. Consequently, there is currently a growing interest in low-cost, efficient xylan-degrading enzymes.

Cellulose enzymatic hydrolysis requires the synergistic action of endo-1,4-β-glucanases (EC 3.2.1.4), *exo*-1,4-β-glucanases or cellobiohydrolases (EC 3.2.1.91), and 1,4-β-glucosidases (EC 3.2.1.21). Endoglucanases randomly cleave internal glycosidic linkages in cellulosic chains, while exoglucanases liberate cellobiose from their edges. β-glucosidases then act on celloligosaccharides and cellobiose, liberating glucose [9]. Since most glucanases are inhibited by cellobiose and short cellooligosaccharides, β-glucosidases catalyze the rate-limiting step of the cellulose hydrolysis process as a whole [15,16]. Owing to its higher structural complexity, xylan saccharification involves two principal enzymes and several accessory ones. *Endo*-β-1,4-xylanases (EC 3.2.1.8) randomly cleave β-1,4-glycosidic linkages in the xylan main chain, releasing xylooligosaccharides, while β-xylosidases (EC 3.2.1.37) release xylose from xylobiose and xylooligosaccharides. Like glucanases, xylanases are also usually inhibited by xylobiose and short xyloligosaccharides, and β-xylosidases are then responsible for the rate-limiting step of xylan hydrolysis. Accessory enzymes, including α-l-arabinofurosidase (EC 3.2.1.55), remove side groups from the xylan main chain [17,18].

Most commercial cellulases and hemicellulases are currently produced by fungi under submerged fermentation. However, solid-state fermentation (SSF) is increasingly being considered an ideal, cost-effective technology for the production of cellulolytic enzymes. SSF conditions mimic the natural environment of fungi, resulting in higher fermentation productivity. Further, cellulases and hemicellulases produced under SSF are often more thermostable and pH-resistant [1,19–21]. On the other hand, low-cost agroindustrial residues may be used as carbon sources, and effluent generation and demands on energy and sterile water are lower. Moreover, the enzymes are obtained at higher concentrations, reducing downstream processing. Since purity is not a pre-requisite for various industrial applications, crude enzyme extracts may then be directly employed [11,19–21]. Altogether, these advantages result in about ten times less production costs, and the optimization of culture conditions may still improve the economic viability of SSF processes [19,21].

The filamentous fungi from the genus *Colletotrichum* and their teleomorph *Glomerella* are recognized as one of the most important plant pathogens distributed on earth, particularly in tropical and subtropical regions. *Colletotrichum* causes plant diseases, usually known as antracnoses. Although different species from this genus are considered models for genetic and physiological studies [22], their potential for enzyme production is unexploited to date. A strain of *Colletotrichum graminicola* was isolated from the Amazon soil, and preliminary studies revealed that it had good potential for the production of β-glucosidases, xylanases and β-xylosidases under SSF, but secreted low levels of cellulases. Thus, crude culture extracts from this strain, supplemented with cellulases, could be interesting for the hydrolysis of lignocellulosic residues, such as SCT.

*Trichoderma reesei* is the best-known cellulase producer among filamentous fungi, and the preferred organism for the production of industrial cellulases [23,24]. The cellulolytic system of *T. reesei* has been extensively investigated. It produces good levels of two cellobiohydrolases (CBH1 and CBH2) and at least four endoglucanases (EG1, EG2, EG3, EG5), but low levels of two β-glucosidases (BGLI and BGLII). Thus, cellulose treatment with *T. reesei* crude culture extracts without an additional load of β-glucosidase results in the accumulation of cellobiose and consequent inhibition of endo- and exo-cellulases, with low glucose yields [15,25].

In this study we optimized the production of β-glucosidase, β-xylosidase and xylanase by a newly isolated strain of *C. graminicola* under SSF in low-cost media constituted of agroindustrial byproducts and/or residues. The efficiency of the crude culture extract from *C. graminicola* alone or in mixture with *T. reesei* crude cellulases for the hydrolysis of sugarcane trash without pre-treatment (RSCT) was also investigated.

## 2. Results and Discussion

### 2.1. Preliminary Screening for the Best Carbon Sources and Supplementary Nitrogen and Carbon Sources for Enzymes Production by *C. graminicola*

The best carbon source for β-glucosidase production was wheat bran (Table 1). The levels of enzyme reached 109.7 ± 7.7 U g^−1^ dry substrate and the fungus showed vigorous growth in this substrate. Good enzyme levels were also obtained in peanut hulls, corn and rice husks. Although the fungus grew poorly in SCT, with a low enzyme production, the supplementation of wheat bran with 1% (*w*/*w*) SCT resulted in 1.6-fold increase in enzyme production levels. Enhanced production (1.4-fold) also resulted from wheat bran supplementation with 1% (*w*/*w*) cellobiose, but filter paper and carboxymethylcellulose (CMC) showed negligible effects. Addition of organic and inorganic nitrogen sources to wheat bran inhibited β-glucosidase production, except for yeast extract and soybean meal, without effect (Table 1).

Maximal levels of β-xylosidase were obtained in wheat bran (57.9 ± 4.6 U g^−1^), but milled corncob, soybean meal and corn husks were also acceptable sources for enzyme production (Table 1). Wheat bran supplementation with 1% peanut hulls increased the production by 42%. Various supplementary carbon sources, including xylan, were somewhat inhibitory, and 1% glucose, xylose or cellobiose reduced the enzyme production by about 40%. Most supplementary nitrogen sources inhibited β-xylosidase production, except for casein and yeast extract, without effect (Table 1).

Wheat bran was also the best carbon source for xylanase production, reaching 189.3 ± 9.5 U g^−1^, and low to negligible enzyme levels were obtained in all other carbon sources (Table 1). Wheat bran supplementation with 1% milled corncob slightly enhanced xylanase production. Most other supplementary carbon sources tested, including xylan, were without effect, while xylose inhibited about 33% the production of the enzyme. The addition of 0.8% (NH_4_)_2_SO_4_ and 1% peptone to wheat bran increased xylanase production (30% and 20%, respectively), while all other nitrogen sources tested inhibited the production, except for casein (Table 1).

Several studies show that wheat bran is a good carbon source for the production of lignocellulolytic enzymes by different fungi under SSF, even in the absence of any supplementary carbon or nitrogen source [26–29]. This has been attributed to its particularly rich nutritional composition: vitamin B, about 14% proteins, 27% carbohydrates (64% cellulose and 36% hemicellulose), 6% lipids, 5% minerals, and around 64% digestible nitrogen [30,31].

### 2.2. Optimization of Enzyme Production by *C. graminicola* Using Response Surface Methodology (RSM)

The optimization of culture media by varying one independent variable at a time maintaining the others at constant levels (OFAT) results in considerable increases in enzyme productivity by microorganisms. However, besides being expensive and time consuming, this methodology does not show the interactions that may occur between different variables that affect enzyme production. In contrast, statistical approaches using response surface methodology not only allow the identification of the optimal conditions by a set of independent variables, but also reveal the interactions between two or more variables [32].

After preliminary studies by OFAT to define the experimental ranges for each independent variable, the culture conditions for the production of β-glucosidase, β-xylosidase and xylanase were optimized using a Central Composite Rotational Design (CCRD).

Data obtained for each enzyme (Tables 2, 3 and 4) were analyzed by multiple regression analysis, and the predicted responses for β-glucosidase (βglu), β-xylosidase (βxyl) and xylanase (Xyl) production were:

(1) $$\beta \text{glu}=182.91-7.39x+11.41y+17.47z-9.45x^{2}-66.88y^{2}-32.69z^{2}$$

(2) $$\beta \text{xyl}=-1732.78+376.92y+359.70z-5.51w-21.76\;y^{2}-48.12z^{2}-3.56w^{2}-4.00yz-5.14wz$$

(3) $$\begin{array}{c} \text{Xyl}=373.94-36.80x+61.96y+91.43z-3.65k-24.45s-195.67x^{2}-113.16y^{2}-112.32z^{2}-\\ 26.04k^{2}-35.44s^{2}+40.33xz \end{array}$$

where *x*, *y*, *z*, *w*, *k* and *s* were the coded values for temperature, culture time, initial moisture, peanut hulls, milled corncob and (NH_4_)_2_SO_4_ concentrations, respectively.

Preliminary analyses by OFAT revealed that the presence of SCT (0.5% to 10%, *w*/*w*) in the culture medium enhanced the production of β-glucosidase by about 60%, compared to that obtained in wheat bran and water only (not shown). However, similar production was obtained for all SCT concentrations tested in this range. In agreement, when the production of β-glucosidase was optimized using RSM the concentration of SCT and its interactions with the other independent variables tested (temperature, culture time and initial moisture) has not significantly influenced the production (not shown). Thus, the concentration of SCT in the culture medium was fixed at 1% for further experiments. Similarly, the temperature and its interactions with the other independent variables tested have not significantly influenced the production of β-xylosidase (not shown), and the production of this enzyme was optimized with respect to culture time, initial moisture and peanut hulls concentration only.

The analyses of variance (ANOVA) for the response surface quadratic models for the production of each enzyme are summarized in Table 5. The coefficients of the full model were analyzed for their significance and the non-significant ones were eliminated from the model. According to the *F* test, the three models were predictive, since the calculated *F* values were greater than the listed *F* values. The low *P*-values also indicate the significance of the models. Further, *P*-values greater than 0.05 indicated that the lack of fit for the models was non-significant. The R^2^ coefficients of 0.99 for β-glucosidase, 0.90 for β-xylosidase and 0.88 for xylanase production confirm the goodness of the three models, indicating that 99%, 90% and 88%, respectively, of the responses variability could be explained by them.

The regression coefficients obtained for β-glucosidase, β-xylosidase and xylanase production are listed in Table 5. The analysis of the *P*-values showed that the quadratic terms of all independent variables tested had significant effects on β-glucosidase production, but not their linear terms nor the interactions between them. The quadratic effects of culture time and initial moisture were the most important factors affecting β-glucosidase production.

Significant effects on β-xylosidase production were exerted by culture time and initial moisture, the interaction between them, and the interaction between peanut hulls concentration and initial moisture. The quadratic terms of all three independent variables also had significant effects. The linear effects of culture time and initial moisture were the most critical variables that affected the production of β-xylosidase.

Analysis of the *P*-values for the regression coefficients obtained for xylanase production revealed that temperature, culture time, initial moisture and (NH_4_)_2_SO_4_ concentration, and their quadratic terms, significantly affected the enzyme production. The quadratic term of milled corncob concentration and the interaction between temperature and initial moisture also had significant effects. The most important factors that affected xylanase production were the quadratic terms of temperature, culture time and initial moisture.

Among the factors that influence the enzymatic production under SSF, water content is well recognized as one of the most critical. The ideal moisture varies with the organism, the operational conditions and the solid substrate, affecting both the microbial growth and the production and secretion of enzymes. At low moisture content, lower substrate swelling occurs, reducing the superficial area. Moreover, the solubility and diffusion of the nutrients are reduced, as well as the stability of the extracellular enzymes, possibly impairing the growth of the microorganism. In contrast, moisture levels that are too high may cause aggregation of substrate particles, reducing the porosity and thus limiting oxygen availability, also hindering microbial growth and lowering enzyme production [28,33,34].

**Figure 1:**
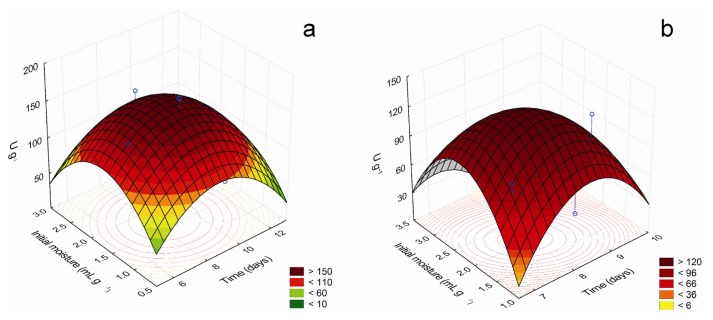
Response surface plots showing the interactive effects of initial moisture and culture time on β-glucosidase (**a**) and β-xylosidase (**b**) production.

**Figure 2:**
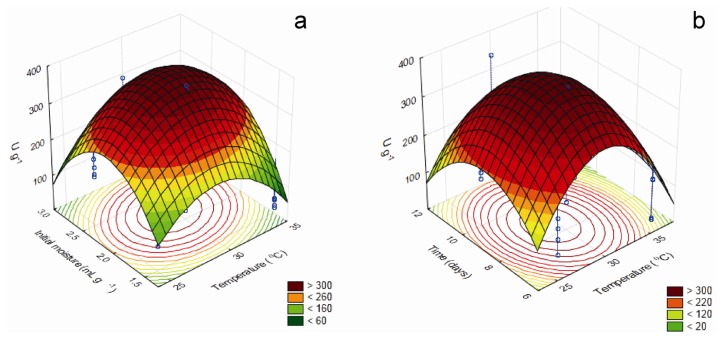
Response surface plots showing the interactive effects of initial moisture and temperature (**a**) and culture time and temperature (**b**) on xylanase production.

Tridimensional response surface curves graphically represent regression equations, demonstrating the relationships between the response and experimental levels of each variable and allowing a better understanding of the interactions between the significant independent variables. Moreover, they provide working ranges for each variable, depicting the robustness of a process, *i.e.*, its capability to bring the expected results in the presence of deviations from the optimal conditions or unexpected adverse factors. The 3D response curves for the most important factors that affect the production of β-glucosidase and β-xylosidase are shown in Figure 1, while Figure 2 brings those for the factors that influence xylanase production.

Statistical analysis showed that maximum β-glucosidase production could be achieved after 7.07 days at 28.13 °C, with initial moisture of 2.38 mL g^−1^, using wheat bran as carbon source and 1% (*w*/*w*) sugarcane trash as supplementary carbon source. Maximum predicted value was 156.63 U g^−1^, 1.4-fold higher than observed before optimization. The mean value of the experimental validation of the optimized conditions (159.3 ± 12.7 U g^−1^) was in excellent correlation with the predicted value, confirming the validity of the model. Interestingly, the production of β-glucosidase under optimal temperature, initial moisture and culture time decreased about 50% in the absence of SCT, but near maximal levels were obtained with SCT concentrations ranging from 1% to 10% (*w*/*w*) (not shown). Considering that SCT does not support the growth of *C. graminicola*, it is possible that when grown in wheat bran, the fungus partially degrades SCT, liberating little amounts of some compounds that stimulate enzyme production.

For β-xylosidase production, the maximum point predicted was 7.71 days, peanut hulls concentration of 4.66% (*w*/*w*) and initial moisture of 2.1 mL g^−1^, at 25 °C, using wheat bran as carbon source. Maximum predicted production was 125.88 U g^−1^, about 2.2-fold higher than that obtained initially. The mean value of the experimental validation was 128.1 ± 6.4 U g^−1^, in good agreement with the predicted value, proving the model’s adequacy.

According to the model, maximum xylanase production occurred at 29.7 °C, 8.66 days, initial moisture of 2.21 mL g^−1^, milled corncob concentration of 0.87% (*w*/*w*) and (NH_4_)_2_SO_4_ concentration of 0.378% (*w*/*w*), using wheat bran as carbon source. The maximal predicted value, 396.65 U g^−1^, was 2.1-fold higher than initially estimated, and showed good correlation with the result of the experimental validation (378.1 ± 23.3 U g^−1^), establishing the model’s validity.

The optimal production levels of β-glucosidase by *C. graminicola* were higher than most reported in the literature for SSF processes, but values of the same magnitude were found for *Aspergillus fumigatus* [35] and *Trichoderma koningi* F244 [36] cultured in wheat bran moistened with mineral salt medium or supplemented with ammonium sulfate, respectively. Similar levels were also obtained for *Thermoascus aurantiacus* grown in wheat straw [37] and *Aspergillus terreus* M11 in corn stover [38], both supplemented with mineral solutions. Few fungi are good producers of β-xylosidases under SSF, but the production levels of the mutant strain *Aspergillus niger* KK2 in rice straw supplemented with salts, yeast extract and corn steep liquor were similar to those found for *C. graminicola* [26]. Higher production levels were reported for a Brazilian strain of *Aspergillus tamarii* cultured in wheat bran supplemented with Vogel salts [39] and for the mutant *Humicola lanuginosa* M7D cultured in corncob supplemented with Vogel salts and corn steep liquor [40]. Similar levels of xylanase, compared to *C. graminicola*, were produced by *A. niger* in wheat bran supplemented with mineral salts [36]. However, 6-fold higher levels of xylanase were produced by some strains of *A. niger* and *T. reesei* [11] in wheat bran supplemented with Mandel Weber medium [41].

Due to the high enzyme loads required for an efficient hydrolysis of lignocellulosic materials, it is estimated that enzyme costs contribute 23%–40% to cellulosic ethanol production cost, directly impacting the economic viability of the process [11,42]. The carbon source is one of the major factors affecting enzyme production costs [38], and thus, there is a rising interest in fungi that produce cellulases and hemicellulases with good yields in inexpensive carbon sources [11,21]. In this context, our results characterize *C. graminicola* as a very promising microorganism for industrial purposes, since few fungal strains produce similar or higher levels of β-glucosidase, β-xylosidase and xylanase under SSF [26,35,38,40]. Moreover, this good production was obtained in quite cheap media mostly composed of wheat bran, a low-cost industrial byproduct, without addition of any expensive nutritional supplement. The simultaneous production of significant levels of three enzymes involved in the hydrolysis of lignocellulose may be considered another advantage of this fungus, aimed at the reduction of production costs. However, it is well established that the hydrolysis of different lignocellulosic residues, which are highly heterogeneous, requires enzymatic cocktails of distinct compositions for maximal yields [8,25]. Thus, the use of multi-enzyme crude extracts has the disadvantage of limiting the possibility of varying enzyme proportions, making difficult to optimize a cocktail for a specific lignocellulosic residue. Moreover, the presence of contaminant enzymes may have deleterious effects for specific industrial processes.

### 2.3. Effect of Temperature and pH on β-Glucosidase, β-Xylosidase and Xylanase Activities and Thermal Stabilities

The effects of pH and temperature on the enzymatic activities in the crude extracts from *C. graminicola* cultured under optimal conditions were evaluated using a CCRD for each enzyme, and RSM analyses. The experimental conditions and results of the experimental designs are summarized in Table 6. Data analyses by multiple regressions led to predicted responses for β-glucosidase (βglu), β-xylosidase (βxyl) and xylanase (Xyl) activities as follows:

(4) $$\beta \text{glu}=-1009.78+225.63\;\text{pH}+15.28\;\text{T}-21.11\;{\text{pH}}^{2}-0.11\;{\text{T}}^{2}-0.26\;\text{pH}.\text{T}$$

(5) $$\beta \text{xyl}=-283.64-63.54\;\text{pH}+5.20\;\text{T}-7.36\;{\text{pH}}^{2}-0.04\;{\text{T}}^{2}+0.11\;\text{pH}.\text{T}$$

(6) $$\text{Xyl}=-675.53+42.76\;\text{pH}+18.32\;\text{T}-3.48\;{\text{pH}}^{2}-0.13\;{\text{T}}^{2}-0.096\;\text{pH}.\text{T}$$

The analyses of variance (ANOVA) for the response surface quadratic models are summarized in Table 7. Based on *F* test, the models were predictive of the enzymatic activities as a function of pH and temperature, given the higher values for calculated *F* values compared to listed ones. The low *p*-values also indicate the models’ significance. Further, the *p*-values greater than 0.05 indicated that the lack of fit for the models was non-significant. The R^2^ coefficients of 0.99 for β-glucosidase, 0.99 for β-xylosidase and 0.98 for xylanase activities confirm the goodness of the models.

**Figure 3:**
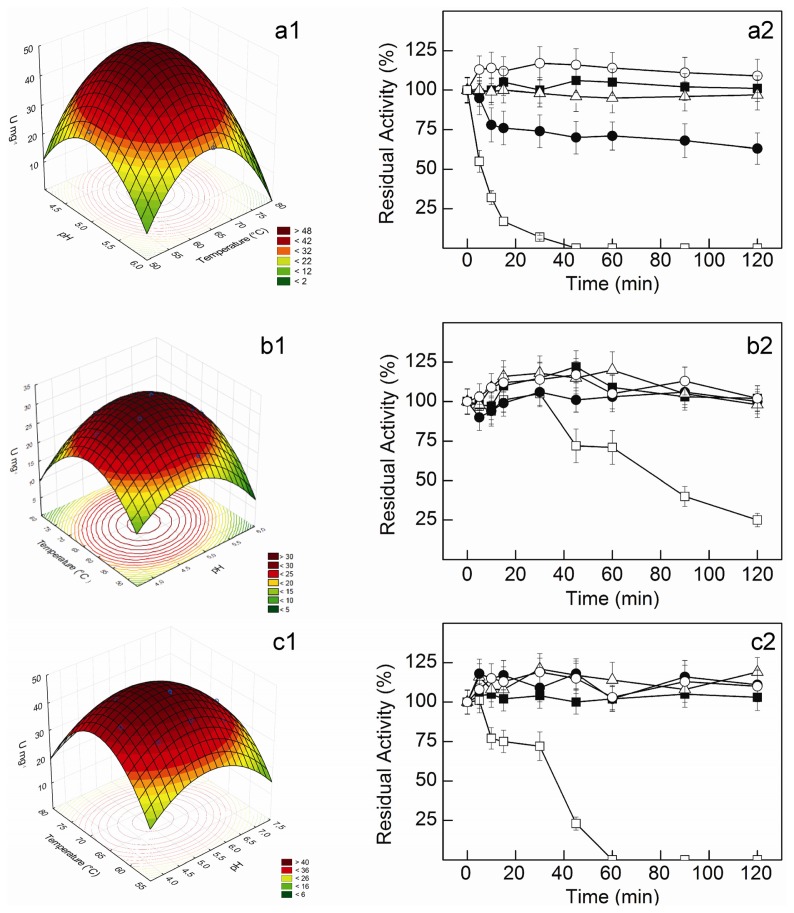
Response surface curves for pH and temperature effects and thermal stabilities of β-glucosidase (**a**), β-xylosidase (**b**) and xylanase (**c**) activities. The fungus was cultured under optimal conditions for the production of each enzyme. For thermal stability studies, aliquots of the crude extracts were incubated at (○) 50 °C, (■) 55 °C, (△) 60 °C, (●) 65 °C or (□) 70 °C. One hundred percent specific activities corresponded to 48.5 ± 1.4 U mg^−1^ for β-glucosidase, 33.2 ± 0.8 U mg^−1^ for β-xylosidase and 47.0 ± 1.4 U mg^−1^ for xylanase. Residual activities were estimated in duplicate aliquots, and the values shown represent averages from triplicate experiments (*n* = 3).

The regression coefficients (Table 7) showed low *p*-values for all linear and quadratic effects, and also for the interaction between pH and temperature, indicating their significant effect on enzymatic activities. The pH effects were higher than the temperature effects for the three activities, and working ranges for near optimal activity may be easily anticipated from the response surface curves for each enzymatic activity (Figure 3). Indeed, this is a clear advantage of using RSM to estimate optimal temperature and pH for enzymatic activity, providing higher flexibility in the development of different bioprocesses [43].

The statistical analysis showed that optimum pH and temperature for β-glucosidase activity were 4.94 and 64.61 °C, and the maximum predicted value was 51.16 U mg^−1^, very close to that obtained in the experimental validation (51.2 ± 1.6 U mg^−1^), confirming the model validity. β-xylosidase activity was maximal at pH 4.77 and 63.33 °C, with a predicted value of 32.59 U mg^−1^, in excellent correlation with the value of experimental validation (32.4 ± 0.8 U mg^−1^). Further, optimal pH and temperature for xylanase activity were 5.21 and 66.91 °C with a predicted value of 49.1 U mg^−1^, in close agreement with the experimental validation, 49.3 ± 1.4 U mg^−1^.

pH optima determined for *C. graminicola* enzymes are in the range found for most fungal β-glucosidases [27,44], β-xylosidases [17] and xylanases [27]. Moreover, their elevated optimal temperatures are highly desirable for industrial applications.

The β-glucosidase from *C. graminicola* maintained about 100% of the initial activity up to 72 h at 50 °C. Moreover, the enzyme was fully stable for 120 min at 55–60 °C. At 65 °C, a rapid decrease to about 80% of the initial activity occurred after 10 min, but a residual activity of 63% remained after 120 min (Figure 3a2). Higher thermal stabilities were observed for the β-xylosidase and the xylanase. Besides retaining full activity for at least 72 h at 50 °C, these enzymes remained stable for 120 min at 55–65 °C (Figure 3b2,c2). At 70 °C, however, half-lives of 6.1, 79.0 and 36.9 min were estimated for β-glucosidase, β-xylosidase and xylanase activities, respectively.

The β-glucosidase from *C. graminicola* was more thermostable than most fungal enzymes studied to date, including those from various thermophilic fungi [44–48]; Few β-glucosidases with higher thermal stabilities were reported [31,49,50]. The β-xylosidase also showed higher thermal stability than reported for enzymes from different thermophilic species [51–56], but somewhat more thermostable enzymes were produced by a few *Aspergillus* genera [17] and *Humicola lanuginosa* [40]. Similarly, *C. graminicola* xylanase was more thermostable than several enzymes from different thermophiles, with half-lives in the range of 4–115 min at 60 °C [57–60]. Some highly thermostable xylanases are, however, produced by *Thermomyces lanuginosus* THKU-49 [61] and *Talaromyces thermophilus* [62], with half-lives of 336 min at 70 °C and 1.0 h at 100 °C, respectively. The high thermal stability of *C. graminicola* β-glucosidase, β-xylosidase and xylanase constitutes another very interesting feature for their employment in biotechnological processes.

### 2.4. Synergic Action of *C. graminicola* Crude Extract and *T. reesei* Cellulases on Raw Sugarcane Trash Hydrolysis

Initially, the optima of temperature and pH, and the thermal stability of the cellulases in the crude extract from *T. reesei* were studied. The optimal pH for cellulase activity was 4.5 to 5.0, and the optimal temperature was 50–55 °C, with a sharp decrease at 60 °C. About 70% of *T. reesei* cellulases activity was retained after 72 h at 50 °C, but at higher temperatures, low residual activities were estimated after 24 h (not shown). Considering that the β-glucosidase, β-xylosidase and xylanase in *C. graminicola* crude extract showed optimal pH around 5.0, where they were fully stable at 50 °C up to 72 h and presented about 55% of their maximal activity at 50 °C, the conditions chosen for RSCT hydrolysis were 50 °C and pH 5.0.

The crude extracts from *T. reesei* and *C. graminicola* were then characterized for specific activities at 50 °C and pH 5.0. *Trichoderma reesei* extract showed good levels of CMCase (3.7 ± 0.5 U mL^−1^), FPA (0.29 ± 0.04 U mL^−1^) and avicelase (0.30 ± 0.05 U mL^−1^), but low levels of cellobiase (0.002 U mL^−1^) and undetectable levels of the other activities tested. In contrast, low levels of cellulases were found in the extract from *C. graminicola* (0.28 ± 0.04 U mL^−1^ CMCase; 0.08 ± 0.02 FPU mL^−1^; 0.044 ± 0.008 U mL^−1^ avicelase), which showed high levels of cellobiase (7.6 ± 0.9 U mL^−1^), β-xylosidase (5.0 ± 0.8 U mL^−1^) and xylanase (34.6 ± 4.9 U mL^−1^) activities, as well as some *α*-L-arabinofuranosidase activity (0.11 ± 0.02 U mL^−1^). Similar profiles were obtained for *C. graminicola* crude extracts produced at optimized conditions for β-glucosidase, β-xylosidase or xylanase production, despite some variation in the proportions of the main enzymes (not shown).

**Figure 4:**
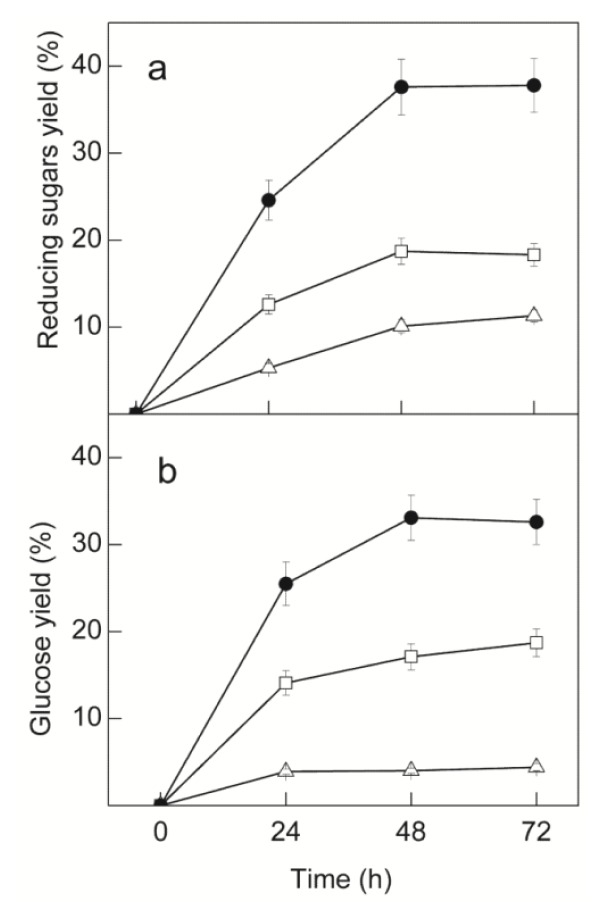
Time course of raw sugarcane trash hydrolysis. (□) *T. reesei* crude extract, enzyme load per gram of substrate 14.5 FPU, 185 U CMCase, 15 U Avicelase and 0.1 U cellobiase; (△) *C. graminicola* crude extract, enzyme load per gram 3.6 FPU, 12.5 U CMCase, 2.0 U Avicelase, 342 U cellobiase, 225 U β-xylosidase, 1557 U xylanase and 5.0 U α-arabinofuranosidase; (●) mixture of *T. reesei* and *C. graminicola* crude extracts, enzyme load per gram 14.6 FPU, 155 U CMCase, 13.6 U Avicelase, 342 U cellobiase, 225 U β-xylosidase, 1557 U xylanase and 5.0 U α-arabinofuranosidase. Enzymatic assays were performed on duplicate aliquots, and the values shown represent averages from triplicate experiments (*n* = 3).

The time course of RSCT hydrolysis using *C. graminicola* and *T. reesei* crude extracts is shown in Figure 4. Treatment with *C. graminicola* extract only resulted in maximal yields of 11.3% reducing sugars and 4.4% glucose after 72 h. Higher yields were obtained using *T. reesei* extract, attaining 18.3% reducing sugars and 18.7% glucose. However, when using a mixture of both extracts, a synergic effect was observed, and yields of 24.6% reducing sugars and 25.5% glucose were attained just after 24 h. Moreover, maximal yields reached 37.6% reducing sugars and 33.1% glucose, after 48 h. The low yields of glucose obtained using *C. graminicola* crude extract reflect its low levels of cellulases. The inefficient hydrolysis of cellulose results in low production of cellobiose and, consequently, low glucose release by β-glucosidases, although present at high levels. The higher levels of reducing sugars compared to glucose thus possibly reflect the action of xylanases and β-xylosidases on RSCT, generating xyloligosaccharides and xylose. In contrast, the reducing sugars obtained using the cellulase-rich crude extract from *T. reesei* must derive from cellulose hydrolysis. Some glucose was also obtained, since this extract presents low levels of β-glucosidases, but the plateau of reducing sugars observed after 48 h suggests accumulation of cellobiose, inhibiting endo- and exo-glucanases, as reported by others [15,25]. The synergic effect observed when a mixture of the two crude extracts was used may be explained by the presence of good levels of xylanases, β-xylosidases and β-glucosidases (*C. graminicola* extract), as well as cellulases (*T reesei* extract), as proposed by other authors [8,10,13,14]. The hydrolysis of xylan by the action of xylanases exposes the cellulosic fibers to cellulases, resulting in higher efficiency of cellulose hydrolysis and liberation of good levels of cellobiose. Efficient cellobiose hydrolysis by β-glucosidases then generates good levels of glucose and releases cellulases from product inhibition. Simultaneously, cellulose degradation increases xylan exposure to xylanases, amplifying the synergic effect. Moreover, the hydrolysis of xylan and xyloligosaccharides to xylose releases the cellulases from inhibition by these compounds [13]. The net result is a faster hydrolysis of RSCT with higher yields of both glucose and reducing sugars. The maximal hydrolysis yield, however, is limited by the high content of lignin in RSCT (20%–36%), that hinders an efficient action of the hydrolytic enzymes [3,42,63,64].

Despite its increasing importance as a sustainable feedstock for cellulosic ethanol production, little information is available on SCT, and few authors have studied its enzymatic hydrolysis. Moreover, most studies reported were carried out using SCT submitted to different pre-treatments to increase the accessibility of cellulose and hemicelluloses to the enzymes [3,42,63–65]. Glucose yields of about 80% to 90% were attained using SCT pre-treated with dilute acid [3,42], steam [63] or ammonia [64], or submitted to milling [65]. In contrast, to the best of our knowledge, a single study provided some data on RSCT hydrolysis [65], reporting a maximal glucose yield of 23.5% after 72 h using a cocktail of commercial enzymes.

The higher efficiency of our cocktail for RSCT hydrolysis, compared to that used by Sant’Ana da Silva *et al.* [65], may be at least in part attributed to the high content of xylanases and β-xylosidases, besides β-glucosidases, in *C. graminicola* crude extract. Similar FPU loads (15 FPU g^−1^ RSCT) were used in both studies. Further, a CMCase load about 1.7-fold lower was employed in this study. In contrast, the cellobiase and xylanase loads were about 3-fold higher, and our cocktail also contained around 225 U g^−1^ of β-xylosidase activity, almost absent (1.4 U g^−1^) from the other cocktail. Thus, our higher glucose yield may be directly related to a higher efficiency of xylan degradation, resulting in an improved action of cellulases. Indeed, the xylan content of SCT is around 23% (*w*/*w*) [5,64], and the composition of *C. graminicola* crude extract seems to be naturally very well suited for the hydrolysis of this particular lignocellulosic residue, with the addition of crude cellulases only.

Finally, considering that the conditions for the hydrolysis of RSCT (incubation time, biomass loading, enzyme loading, *etc.*) using the *C. graminicola* cocktail were not yet optimized, increases in glucose yields are to be expected. Moreover, the addition of some other enzymes to the cocktail, such as ligninases, may increase the hydrolysis efficiency, as well as some mild, low-cost pre-treatment of SCT. Thus, this low-cost cellulolytic-hemicelulolytic enzyme system seems promising for future industrial use. However, its real practical applicability depends on several factors, including the success of scaling up the processes of enzyme production and SCT hydrolysis. Further, the implementation of any industrial process of enzymatic hydrolysis of a lignocellulosic residue for ethanol production depends on a complete techno-economic evaluation of the final production costs [5].

## 3. Experimental Section

### 3.1. Organisms and Strains Maintenance

The *Colletotrichum* strain was isolated by chance from Amazon rainforest soil, Brazil. Samples from soil were cultured in PDA supplemented with 50 μg·mL^−1^ antibiotics from Fort Dodge (Brazil) which contains a mixture of benzylpenicillin benzathine, benzylpenicillin procaine, diidroestreptomicin sulfate, potassium benzylpenicillin and streptomycin sulfate. The cultures were incubated at 25 °C for 48–72 h and the initial colonies formed were transferred and maintained in laboratory in PDA slant medium. *Colletotrichum graminicola* was classified on basis of physiological and morphological characteristics, including the conidiophores morphology, analyzed according to Barnett and Hunter [66] and confirmed by conidia germination analyses [67]. The fungus was maintained on Potato Dextrose Agar (PDA: Oxoid, UK) medium, at 25 °C. *Trichoderma reesei* QM9414 (ATCC 26921) was maintained at 25 °C on solid 4% oatmeal baby food (Quaker) medium.

### 3.2. Preliminary Screening for the Best Carbon Sources and Supplementary Nitrogen and Carbon Sources for the Production of β-Glucosidase, β-Xylosidase and Xylanase

Sections (0.25 cm^2^) of the mycelium of *C. graminicola* grown on PDA medium were taken from 8-day-old cultures and inoculated into sterile solid media prepared in 250 mL Erlenmeyer flasks, containing 10 mL of deionized water and 5 g of different dry carbon sources. Each section contained fungi in all extension and was put onto the culture medium using an inoculation needle, in order to achieve the maximum contact between the mycelium and the medium. The transferences of the mycelial sections were performed in an inoculation chamber, and all the usual aseptic procedures were maintained both during the transferences and the cultures growth. The flasks were incubated for 192 h under static conditions in a controlled environment incubator (New Brunswick Scientific: Edison, NJ, USA) at 25 °C and 70% humidity, monitored by a thermo hygrometer (Minipa: São Paulo State, Brazil). All experiments were conducted in triplicate. The enzymatic assays were carried out in duplicate. After choosing the best carbon source for the production of each enzyme, the effect of supplementation with different nitrogen and carbon sources was investigated under the same culture conditions.

### 3.3. Response Surface Methodology for the Optimization of β-Glucosidase, β-Xylosidase and Xylanase Production

After selecting the best carbon source, and supplementary nitrogen and carbon sources for the production of each enzyme, RSM was employed for production optimization.

To optimize the production of β-glucosidase using wheat bran as the carbon source, the levels of the independent variables (sugarcane trash concentration, temperature, culture time, medium initial moisture) were defined according to a 2^4^ full-factorial central composite design (star configuration) with 8 axial and 3 central points (triplicate in the central point only) which resulted in 27 experiments. The experimental ranges of each independent variable tested in RSM experiments were determined preliminarily, using the OFAT methodology, corresponding to a sugarcane trash concentration range of 0.5% to 10% (*w*/*w*), a temperature range of 20–40 °C, culture time from 7 to 11 days and medium initial moisture from 1.0 to 2.5 mL deionized H_2_O g^−1^ dry substrate (wet basis moisture content from 46.9% to 73%, *w*/*w*). Since SCT concentration and its interactions with the other independent variables tested have not significantly influenced enzyme production, SCT was added to wheat bran at 1% (*w*/*w*) fixed concentration. The levels of the other independent variables (temperature, culture time, medium initial moisture) were then defined according to a 2^3^ full-factorial central composite design (star configuration) with six axial and two central points (duplicate in the central point only) which resulted in 17 experiments (Table 2).

Similarly, to optimize the production of β-xylosidase, the levels of the independent variables (concentration of peanut hull, temperature, culture time, medium initial moisture) were defined according to a 2^4^ full-factorial central composite design (star configuration) with 8 axial and 3 central points (triplicate in the central point only) which resulted in 27 experiments. The experimental ranges of each independent variable tested were defined preliminarily using the OFAT methodology, corresponding to a peanut hulls concentration range of 0.5% to 7% (*w*/*w*), a temperature range of 20–40 °C, culture time from 6 to 10 days and medium initial moisture from 0.5 to 3.0 mL deionized H_2_O per g dry substrate (wet basis moisture content from 31.3% to 73%, *w*/*w*). Since temperature and its interactions with the other independent variables tested have not significantly influenced enzyme production, it was fixed at 25 °C. The levels of the other independent variables (concentration of peanut hull, culture time, medium initial moisture) were defined according to a 2^3^ full-factorial central composite design (star configuration) with six axial and three central points (triplicate in the central point only) which resulted in 17 experiments (Table 3).

For xylanase production optimization, the levels of the independent variables (temperature, culture time, medium initial moisture, milled corncob concentration and ammonium sulfate concentration) were defined according to a 2^5−1^ fractional factorial central composite design (star configuration) with ten axial and six central points (six replicates of the central point only), resulting in 32 experimental runs (Table 4). The experimental ranges of each independent variable tested were defined preliminarily using the OFAT methodology, corresponding to: temperature, 20–40 °C; culture time, 4–12 days; medium initial moisture, 1.0–3.0 mL deionized H_2_O per g dry substrate (wet basis moisture content from 46.9% to 73%, *w*/*w*), milled corncob concentration, 0.0%–2.0%, *w*/*w*; ammonium sulfate concentration, 0.1%–0.9% (*w*/*w*). All the experiments were conducted in triplicate and the enzymatic assays were carried out in duplicate. Initial moisture of the culture media was expressed as volume of deionized H^2^O (mL) per g dry substrate or as wet basis moisture content, experimentally determined and calculated according to the following equation:

(7) $$M_{wb}=\frac{m_{H_{2}O}}{m_{H_{2}O}+m_{ds}}$$

where *M*_wb_ (%) = wet-basis moisture content; *m*_H_2_O_ = mass of moisture (g), and *m*_ds_ = mass of dry substrate (g).

### 3.4. Enzyme Extraction

After *C. graminicola* growth, culture medium was suspended in 30 mL cold water, gently homogenized with a glass rod in an ice bath, filtered through a nylon sieve and centrifuged at 10,000× *g* and 4 °C for 15 min. The supernatant (crude extract) was maintained at 4 °C with no appreciable losses of β-glucosidase, β-xylosidase and xylanase activities up to 30 days.

### 3.5. Enzymatic Assays

β-glucosidase and β-xylosidase activities were routinely determined using p-nitrophenyl-β-d-glucopyranoside and p-nitrophenyl-β-d-xylopyranoside (pNP-glu and pNP-xyl, respectively, Sigma-Aldrich Chem. Co.: St. Louis, MO, USA), as described by Souza *et al.* [44]. Standard assay conditions were 65 °C, 50 mmol L^−1^ sodium acetate buffer, pH 5.0, and 2.0 mmol L^−1^ substrate, in a final volume of 0.6 mL. Xylanase activity was routinely assayed in the same conditions using 1% (*m*/*v*) Birchwood xylan (Sigma-Aldrich Chem. Co.: St. Louis, MO, USA) as substrate, in a final volume of 1.0 mL. The reducing sugars released were quantified using the dinitrosalicylic acid (DNS) method [68].

To characterize the crude extracts from *C. graminicola* and *T. reesei* employed in RSCT hydrolysis, β-glucosidase, β-xylosidase and xylanase activities were assayed as above, at 50 °C. α-l-Arabinofurosidase activity was assayed in the same conditions using p-nitrophenyl-α-L-arabinofuranoside (Sigma-Aldrich Chem. Co.: St. Louis, MO, USA) as substrate, according to the method described by Souza *et al.* [44]. Hydrolysis of cellobiose (10 mmol L^−1^) was estimated under the same conditions by quantifying glucose released using the glucose oxidase method [69]. Hydrolytic activities against microcrystalline cellulose (Avicel^®^, Fluka Chemical Co.: Seelze, Germany), carboximethylcellulose (CMC) and filter paper (FPase activity) were assayed in the same conditions using Avicel or CMC at 1% (*w*/*v*) concentration, or a strip (1 cm × 3 cm) of Whatman No. 1 filter paper, quantifying the reducing sugars released by the DNS method.

Experimental conditions (reaction times, enzymatic units) employed in all activity measurements were adjusted to guarantee the estimation of initial velocities (hydrolysis of no more than 5% initial substrate, linear response of product formation in respect to reaction time). One enzyme unit (1U) was defined as the amount of enzyme that releases 1 μmol of product per min. One filter paper enzyme unit (FPU) was defined as the amount of enzyme that releases 1 μmol of product per min using filter paper as substrate.

### 3.6. Effect of Temperature and pH on Enzymatic Activities and Thermal Stabilities

To evaluate the effects of temperature and pH and their possible interactions on β-glucosidase activity of *C. graminicola* crude extract the levels of the independent variables were defined according to a 2^2^ full-factorial central composite design (star configuration) comprising 11 experimental runs, including 4 axial and 3 central points (triplicate in the central point only). The same design was applied for β-xylosidase and xylanase activities (Table 6). The reactions were carried out using 2 mmol L^−1^ pNP-glu or pNP-xyl or 1% xylan as substrates in McIlvaine buffer [70] at different pH values. All the experiments were conducted in quadruplicate and the enzymatic assays were carried out in triplicate.

The optimum temperature for cellulase activity of *T. reesei* crude extract was determined in the range from 35 to 60 °C in 50 mmol L^−1^ sodium acetate buffer, pH 5.0, using CMC, filter paper and avicel as substrates. The optimum pH for the hydrolysis of each substrate was determined at 50 °C in McIlvaine buffer [70] at different pH (3.0 to 8.0).

The thermal stability of *C. graminicola* β-glucosidase, β-xylosidase and xylanase was evaluated by incubating the crude extract for different time intervals at temperatures ranging from 50 to 70 °C. After cooling in an ice bath for 1 min, the residual activities were determined at 65 °C. The thermal stability of CMCase activity of *T. reesei* crude extract was evaluated as above, at 50 °C, and the residual activity was estimated at 50 °C. The enzymatic activity assays using different substrates were carried out as described in the item 3.5.

### 3.7. Determination of Protein

Protein concentrations were measured according to Read and Northcote [71] using bovine serum albumin as standard.

### 3.8. Data Fitting and Statistical Analysis

RSM was conducted using the software Statistica, version 10.0 (StatSoft, Inc.: Tulsa, OK, USA) for the experimental designs and regression analyses of the data. A statistical analysis of each model was performed to estimate the analysis of variance (ANOVA), the quality of each polynomial model equation was statistically evaluated by the coefficient *R*^2^, and its statistical significance was checked by an F-test. For all other experiments, data fitting and statistical analyses were carried out using OriginPro 8 SRO software package (OriginLab Corp.: Northampton, MA, USA).

### 3.9. Experimental Validation of the Optimized Conditions

Three cultures for β-glucosidase, β-xylosidase and xylanase production, under optimized conditions, were carried out to validate the optimization. Similarly, the optimization of reaction pH and temperature for the enzymatic activities was validated by performing a triplicate experiment in the optimized conditions.

### 3.10. Synergic Action of *C. graminicola* and *T. reesei* Crude Extracts on RSCT Hydrolysis

*Colletotrichum graminicola* crude extract was obtained from 9-day cultures at 30 °C in wheat bran containing 0.9% (*w/w*) milled corncob and 0.4% (*w*/*w*) (NH_4_)_2_SO_4_, with initial moisture of 2.2 mL g^−1^ (optimal conditions for xylanase production), at 70% humidity. *Trichoderma reesei* QM9414 was grown for 72 h at 28 °C and 130 rpm in 500-mL Erlenmeyer flasks containing 100 mL of liquid medium composed of 0.03% urea, 0.14% (NH_4_)_2_SO_4_, 0.2% KH_2_PO_4_, 0.03% CaCl_2_·2H_2_O, 0.03% MgSO_4_·7H_2_O, 5.0 mg mL^−1^ FeSO_4_·7H_2_O, 1.6 mg mL^−1^ MnSO_4_, 1.4 mg mL^−1^ ZnSO_4_. 7H_2_O, 2 mg mL^−1^ CoCl_2_, 0.075% peptone, 0.2% Tween-80 and 0.75% Avicel^®^, with initial pH adjusted for 5.5–6.0. Cellulase-rich *T. reesei* crude extract, obtained by filtration of the culture medium through synthetic sponge, was maintained at 4 °C for a month without appreciable activity losses.

Before hydrolysis, RSCT (kindly provided by Companhia Albertina Mercantil e Industrial: Sertãozinho, São Paulo State, Brazil) was washed until detection of negligible levels of reducing sugars in washing waters (DNS method). Washed RSCT was dried overnight at 60 °C and grinded with a domestic blender (5 min, maximum speed). Enzymatic hydrolysis was carried out at 50 °C and 180 rpm in 50 mmol L^−1^ sodium acetate buffer, pH 5.0, containing 1% (*w*/*v*) RSCT and 10 mmol L^−1^ sodium azide in a final volume of 20 mL, in hermetically closed 250-mL Erlenmeyer flasks. Hydrolysis was initiated by addition of an aliquot of *T. reesei* crude extract, an aliquot of *C. graminicola* crude extract or a mixture of both. After desired time intervals, 0.5 mL aliquots of the reaction medium were withdrawn, centrifuged at 10,000× *g* for 5 min and the supernatants were analyzed for reducing sugars and glucose.

The yield of RSCT hydrolysis was calculated as a percentage of maximal reducing sugars or glucose released by sulfuric acid hydrolysis, according to the National Renewable Energy Laboratory (NREL) Laboratory Analytical Procedure (LAP) for the quantification of structural carbohydrates [72].

## 4. Conclusions

*Colletotrichum graminicola* is a significant producer of β-glucosidase, β-xylosidase and xylanase under SSF in cheap media, comprising of wheat bran as carbon source and agricultural residues as supplements. All three enzymes are produced simultaneously at good levels, and crude culture extracts may thus be used without further processing to compose efficient cocktails for lignocellulosic material hydrolysis. Indeed, a cocktail composed of *C. graminicola* crude extract supplemented with *T. reesei* crude cellulases hydrolyzed raw sugarcane trash with a yield of 33.1% glucose after 48 h, demonstrating strong potential for the hydrolysis of this interesting, cheap feedstock for the production of cellulosic ethanol.

## Figures and Tables

**Table 1:** Effect of different carbon sources on β-glucosidase, β-xylosidase and xylanase production by *C. graminicola* under solid-state fermentation.

Carbon Source		β-glucosidase (U g^−1^)		β-xylosidase (U g^−1^)		Xylanase (U g^−1^)	
Wheat bran		109.7 ± 7.7		57.9 ± 4.6		189.3 ± 9.5	
Steam-exploded sugarcane bagasse		9.5 ± 1.1		15.9 ± 1.4		ND a	
Raw sugarcane bagasse		20.8 ± 1.8		6.9 ± 0.8		11.4 ± 1.1	
Sugarcane trash		10.2 ± 1.2		11.5 ± 1.3		15.7 ± 1.6	
Peanut hull		57.0 ± 4.1		9.4 ± 1.2		6.4 ± 0.7	
Rice husk		47.5 ± 4.1		13.6 ± 1.5		9.6 ± 1.2	
Corn husk		48.3 ± 3.9		24.7 ± 2.4		ND	
Milled corncob		-		37.8 ± 3.3		15.7 ± 1.8	

**Carbon Supplement (1%*w*/*w*)**		**β-glucosidase**		**β-xylosidase**		**Xylanase**	

		**U g^−1^**	**%**	**U g^−1^**	**%**	**U g^−1^**	**%**

None		101.1 ± 7.8	100.0	55.5 ± 5.1	100	193.2 ± 13.8	100
Peanut hull		49.2 ± 4.6	48.7	79.0 ± 7.7	142.3	178.0 ± 11.3	92.1
Rice husk		33.6 ± 3.2	33.2	49.4 ± 5.1	89.0	176.1 ± 12.3	91.1
Corn husk		54.9 ± 5.3	54.0	38.8 ± 3.9	69.9	205.6 ± 14.3	106.4
Milled corncob		-	-	47.5 ± 4.8	85.6	239.8 ± 15.2	124.1
Sugarcane trash		162.9 ± 13.1	161.1	40.2 ± 4.1	72.4	178.4 ± 10.9	92.3
Filter paper		97.9 ± 8.4	96.8	43.9 ± 3.8	79.1	189.5 ± 12.9	98.1
Glucose		91.4 ± 8.4	90.4	35.5 ± 4.3	64.0	231.8 ± 15.3	120.0
Xylose		-	-	34.7 ± 4.5	62.5	129.5 ± 10.4	67.0
Celobiose		144.8 ± 10.4	143.2	34.0 ± 4.2	61.3	173.4 ± 14.9	89.7
CMC		84.8 ± 7.6	83.9	48.3 ± 4.2	87.0	171.6 ± 13.4	88.8
Xylan		-	-	42.1 ± 3.9	75.8	204.5 ± 15.4	105.8

**Nitrogen Supplement**	**% (*w*/*w*)**	**β-glucosidase**		**β-xylosidase**		**Xylanase**	

		**U g^−1^**	**%**	**U g^−1^**	**%**	**U g^−1^**	**%**

None	-	92.5 ± 8.9	100.0	47.8 ± 5.2	100	152.7 ± 12.2	100
Asparagine	1	42.4 ± 5.1	45.8	38.8 ± 4.2	81.2	118.5 ± 10.7	77.6
Glycine	1	35.4 ± 4.6	38.3	37.1 ± 4.3	77.6	124.2 ± 11.0	81.3
Casein	1	39.7 ± 4.8	42.9	47.4 ± 5.1	99.1	151.3 ± 11.7	99.1
Peptone	1	40.4 ± 5.2	43.7	36.1 ± 4.0	75.5	182.7 ± 13.7	119.6
Yeast extract	1	88.4 ±7.9	95.6	43.9 ± 4.8	91.8	164.1 ± 12.8	107.5
Malt extract	1	41.6 ± 4.6	45.0	27.2 ± 3.4	56.9	129.9 ± 11.2	85.1
Soybean meal	1	87.5 ± 9.6	94.6	37.7 ± 4.2	78.9	127.0 ± 11.6	83.2
Urea	0.8	39.9 ± 3.2	43.1	19.5 ± 2.8	40.8	97.1 ± 8.7	63.6
NH_4_NO_3_	0.8	40.9 ± 3.7	44.2	23.9 ± 3.1	50.0	125.6 ± 11.3	82.2
KNO_3_	0.8	41.7 ± 4.2	45.1	21.1 ± 2.6	44.1	131.3 ± 11.0	86.0
NaNO_3_	0.8	38.9 ± 4.3	42.0	26.5 ± 3.1	55.4	122.7 ± 10.2	80.3
(NH_4_)_2_SO_4_	0.8	44.7 ± 4.0	48.3	23.7 ± 2.7	49.6	198.4 ± 13.5	129.9
NH_4_Cl	0.8	40.7 ± 3.7	44.0	32.9 ± 3.6	68.8	125.6 ± 11.3	82.2

Notes: *Colletotrichum graminicola* was cultured for 192 h at 25 °C in 5 g dry carbon source (or a mass occupying maximally 1/5 of the total volume of the culture flask) and deionized water (2 mL g^−1^ dry substrate). Supplementary nitrogen and carbon sources were added to wheat bran as carbon source at the indicated concentrations. Enzymatic assays were performed in duplicate; each experiment was repeated using four different crude extracts (*n* = 4). Data are presented as means ± SD;

a ND: undetectable by the methods used.

**Table 2:** Experimental conditions and results of the statistical experimental design for β-glucosidase production by *C. graminicola*.

Run	Real(Coded) values			

	*x*	*y*	*z*	β-glucosidase a (U g^−1^)
**1**	35(1)	8(−1)	1.5(−1)	118.0
**2**	30(0)	9(0)	1.5(−1)	150.6
**3**	20(−1.68)	9(0)	2.0(0)	102.0
**4**	25(−1)	8(−1)	2.5(1)	146.0
**5**	40(+1.68)	9(0)	2.0(0)	80.0
**6**	30(0)	9(0)	2.0(0)	171.0
**7**	35(1)	10(1)	1.5(−1)	114.0
**8**	25(−1)	10(1)	2.5(1)	111.4
**9**	25(−1)	8(−1)	1.5(−1)	100.0
**10**	35(−1)	10(1)	2.5(1)	119.2
**11**	30(0)	9(0)	3.0(+1.68)	164.0
**12**	35(1)	8(−1)	2.5(1)	114.0
**13**	30(0)	6(−1.68)	2.0(0)	140.0
**14**	30(0)	11(+1.68)	2.0(0)	135.0
**15**	25(−1)	10(1)	1.5(−1)	100.6
**16**	30(0)	9(0)	1.0(−1.68)	99.0
**17**	30(0)	9(0)	2.0(0)	171.8

Notes: *x*: temperature (°C); *y*: time (days); *z*: initial moisture (mL g^−1^). Initial moisture of the culture media expressed as wet basis moisture content corresponded to: 46.9% (1 mL g^−1^), 55.8% (1.5 mL g^−1^), 63.5% (2.0 mL g^−1^), 68.6% (2.5 mL g^−1^) and 73.0% (3.0 mL g^−1^);

a Response values for each run were the means of triplicate experiments, and each enzymatic assay was carried out in duplicate.

**Table 3:** Experimental conditions and results of the statistical experimental design for β-xylosidase production by *C. graminicola*.

Run	Real(Coded) values			

	*w*	*y*	*z*	β-xylosidase a (U g^−1^)
**1**	1(−1)	7(−1)	1.5(−1)	93.8
**2**	5(1)	7(−1)	1.5(−1)	85.6
**3**	1(−1)	7(−1)	1.5(−1)	99.6
**4**	5(1)	7(−1)	1.5(−1)	104.2
**5**	1(−1)	9(1)	2.5(1)	76.8
**6**	5(1)	9(1)	1.5(−1)	72.6
**7**	1(−1)	9(1)	2.5(1)	60.6
**8**	5(1)	9(1)	2.5(1)	85.4
**9**	3(0)	6(−1.68)	2.0(0)	56.0
**10**	3(0)	11(+1.68)	2.0(0)	58.6
**11**	3(0)	8(0)	1.0(−1.68)	59.6
**12**	3(0)	8(0)	3.0(+1.68)	86.0
**13**	0.5(−1.68)	8(0)	2.0(0)	40.2
**14**	7(+1.68)	8(0)	2.0(0)	117.4
**15**	3(0)	8(0)	2.0(0)	126.0
**16**	3(0)	8(0)	2.0(0)	123.0
**17**	3(0)	8(0)	2.0(0)	124.6

Notes: *w*: peanut hulls (%, *w/w*); *y*: time (days); *z*: initial moisture (mL g^−1^). Initial moisture of the culture media expressed as wet basis moisture content corresponded to: 46.9% (1 mL g^−1^), 55.8% (1.5 mL g^−1^), 63.5% (2.0 mL g^−1^), 68.6% (2.5 mL g^−1^) and 73.0% (3.0 mL g^−1^).

a Response values for each run were the means of triplicate experiments, and each enzymatic assay was carried out in duplicate.

**Table 4:** Experimental conditions and results of the statistical experimental design for xylanase production by *C. graminicola*.

Run	Real(Coded) Values					

	*x*	*y*	*z*	*k*	*s*	Xylanase (U g^−1^)
**1**	25(−1)	6(−1)	1.5(−1)	0.5(−1)	0.7(1)	110.6
**2**	25(−1)	6(−1)	1.5(−1)	1.5(1)	0.3(−1)	70.9
**3**	25(−1)	6(−1)	2.5(1)	0.5(−1)	0.3(−1)	166.3
**4**	25(−1)	6(−1)	2.5(1)	1.5(1)	0.7(1)	140.4
**5**	25(−1)	10(1)	1.5(−1)	0.5(−1)	0.3(−1)	161.0
**6**	25(−1)	10(1)	1.5(−1)	1.5(1)	0.7(1)	128.9
**7**	25(−1)	10(1)	2.5(1)	0.5(−1)	0.7(1)	146.8
**8**	35(1)	10(1)	2.5(1)	1.5(1)	0.3(−1)	190.5
**9**	35(1)	6(−1)	1.5(−1)	0.5(−1)	0.3(−1)	39.1
**10**	35(1)	6(−1)	1.5(−1)	1.5(1)	0.7(1)	43.2
**11**	35(1)	6(−1)	2.5(1)	0.5(−1)	0.7(1)	146.8
**12**	35(1)	6(−1)	2.5(1)	1.5(1)	0.3(−1)	156.9
**13**	35(1)	10(1)	1.5(−1)	0.5(−1)	0.7(1)	16.4
**14**	35(1)	10(1)	2.5(−1)	0.5(−1)	0.3(−1)	22.8
**15**	35(1)	10(1)	2.5(1)	0.5(−1)	0.3(−1)	197.9
**16**	35(1)	10(1)	2.5(1)	1.5(1)	0.7(1)	122.3
**17**	20(−2)	8(0)	2.0(0)	0(1.0)	0.5(0)	32.3
**18**	40(2)	8(0)	2.0(0)	1.0(0)	0.5(0)	0.6
**19**	30(0)	4(−2)	2.0(0)	1.0(0)	0.5(0)	25.2
**20**	30(0)	12(2)	2.0(0)	1.0(0)	0.5(0)	337.1
**21**	30(0)	8(0)	1.0(−2)	1.0(0)	0.5(0)	75.4
**22**	30(0)	8(0)	3.0(2)	1.0(0)	0.5(0)	290.2
**23**	30(0)	8(0)	2.0(0)	0.0(−2)	0.5(0)	370.5
**24**	30(0)	8(0)	2.0(0)	2.0(2)	0.5(0)	373.5
**25**	30(0)	8(0)	2.0(0)	1.0(0)	0.1(−2)	375.8
**26**	30(0)	8(0)	2.0(0)	1.0(0)	0.9(2)	299.0
**27**	30(0)	8(0)	2.0(0)	1.0(0)	0.5(0)	352.8
**28**	30(0)	8(0)	2.0(0)	1.0(0)	0.5(0)	337.3
**29**	30(0)	8(0)	2.0(0)	1.0(0)	0.5(0)	364.7
**30**	30(0)	8(0)	2.0(0)	1.0(0)	0.5(0)	355.7
**31**	30(0)	8(0)	2.0(0)	1.0(0)	0.5(0)	349.9
**32**	30(0)	8(0)	2.0(0)	1.0(0)	0.5(0)	361.1

Notes: *x*: temperature (°C); *y*: time (days); *z*: initial moisture (mL g^−1^); *k*: milled corncob (%, *w*/*w*); *s*: ammonium sulfate concentration (%, *w*/*w*). Initial moisture of the culture media expressed as wet basis moisture content corresponded to: 46.9% (1 mL g^−1^), 55.8% (1.5 mL g^−1^), 63.5% (2.0 mL g^−1^), 68.6% (2.5 mL g^−1^) and 73.0% (3.0 mL g^−1^).

a Response values for each run were the means of triplicate experiments, and each enzymatic assay was carried out in duplicate.

**Table 5:** Analysis of variance (ANOVA) for the second order polynomial models, and coefficient values for β-glucosidase, β-xylosidase and xylanase production by *C. graminicola*.

Source	β-glucosidase					β-xylosidase					Xylanase				

	df	SS	MS	*F*	*p*	df	SS	MS	*F*	*p*	df	SS	MS	*F*	*p*
**M**	9	14,792.38	1,578.15	7.08	0.0039	9	8,927.015	991.89	6.17	0.0191	20	402,029.4	20,101.47	3.40	0.0402
**R**	8	1,452.74	165.89			6	964.563	160.76	-	-	8	47,279.11	5,909.89	-	-
**LF**	5	1,093.82	179.63	1.94	0.0643	6	1,441.22	240.204	99.258	0.0766	7	53,966.3	3,597.8	19.12	0.0507
**PE**	3	3.17	10.3	-	-	1	2.42	0.5	-	-	2	376.3	188.2	-	-

**Effects**			**Coefficient**	***p*-value**		**Effects**		**Coefficient**	***p*-value**		**Effects**		**Coefficient**	***p*-value**	

Intercept			182.19	0.0009 c		Intercept		−1732.78	0.0146 a		Intercept		373.94	0.0004 c	
*X*			−7.39	0.1985		*w*		−5.51	0.2404		*x*		−36.80	0.0223 a	
*x^2^*			−9.45	0.0198 a		*w*^2^		−3.56	0.0202 a		*x^2^*		−195.67	0.0008 c	
*Y*			11.41	0.1429		*y*		376.92	0.0144 a		*y*		61.96	0.0080 a	
*y^2^*			−66.88	0.0009 a		*y^2^*		−21.76	0.0015 b		*y^2^*		−113.16	0.0023 b	
*Z*			17.47	0.0667		*z*		359.70	0.0185 a		*z*		91.43	0.0037 b	
*z^2^*			−32.69	0.0087 a		*z^2^*		−48.12	0.0196 a		*z^2^*		−112.32	0.0024 b	
-			-	-		*wz*		5.14	0.0339 a		*k*		−3.65	0.5807	
-			-	-		*yz*		4.00	0.0416 a		*k^2^*		−26.04	0.0423 a	
-			-	-							*s*		−24.45	0.0486 a	
											*s^2^*		−35.44	0.0235 a	
-			-	-		-		-	-		*xz*		40.33	0.0277 a	

Notes: β-glucosidase: R^2^ = 0.99; *F* listed 5% = 3.35; β-xylosidase: R^2^ = 0.90; *F* listed 5% = 3.22; Xylanase: R^2^ = 0.88; *F* listed 5% = 2.64, M = Model; R = residual; LF = Lack of fit; PE = Pure error; df: degrees of freedom; SS: sum of squares; MS: mean square. *x*: temperature (°C); *y*: time (days); *z*: initial moisture (mL g^−1^); *w*: peanut hulls concentration (%, *w*/*w*); *k*: milled corncob (%, *w*/*w*); *s*: ammonium sulfate concentration (%, *w*/*w*).

a *p* ≤ 0.05;

b *p* ≤ 0.005;

c *p* ≤ 0.001

**Table 6:** Experimental conditions and results of the statistical experimental designs for β-glucosidase, β-xylosidase and xylanase activities.

Run	β-glucosidase a			β-xylosidase a			Xylanase a		

	pH	T (°C)	U mg^−1^	pH	T (°C)	U mg^−1^	pH	T (°C)	U mg^−1^
**1**	4.5(−1)	55(−1)	35.7	4.5(−1)	55(−1)	31.5	4.5(−1)	60(−1)	40.3
**2**	6.0(+1.41)	65(0)	27.6	6.0(+1.41)	65(0)	21.7	4.5(−1)	70(1)	46.2
**3**	5.5(1)	55(−1)	35.6	5.5(1)	55(−1)	27.7	6.5(1)	60(−1)	37.6
**4**	4.0(−1)	75(1)	36.3	4.5(−1)	75(1)	24.6	6.5(1)	70(1)	41.6
**5**	5.5(1)	75(1)	30.9	5.5(1)	75(1)	23.5	4.0(−1.41)	65(0)	43.4
**6**	5.0(0)	65(0)	51.1	5.0(0)	65(0)	32.3	7.0(+1.41)	65(0)	37.0
**7**	5.0(0)	50(−1.41)	27.8	5.0(0)	50(−1.41)	24.0	5.5(0)	55(−1.41)	48.3
**8**	5.0(0)	65(0)	51.2	5.0(0)	65(0)	31.1	5.5(0)	75(+1.41)	48.4
**9**	4.0(−1.41)	65(0)	32.5	4.0(−1.41)	65(0)	28.0	5.5(0)	65(0)	48.2
**10**	5.0(0)	80(+1.41)	24.9	5.0(0)	80(+1.41)	20.0	5.5(0)	65(0)	48.4
**11**	5.0(0)	65(0)	50.2	5.0(0)	65(0)	31.9	5.5(0)	65(0)	48.7

Note:

a Response values for each run were the means of four experiments, and each enzymatic assay was carried out in triplicate.

**Table 7:** Analysis of variance (ANOVA) for the second order polynomial models, and coefficient values for β-glucosidase, β-xylosidase and xylanase activities in *C. graminicola* crude extract.

Source	β-glucosidase					β-xylosidase					Xylanase				

	df	SS	MS	*F*	*p*	df	SS	MS	*F*	*p*	df	SS	MS	*F*	*p*
**M**	5	980.468	196.094	46.11	0.0002	5	202.638	40.528	149.06	0.0003	5	187.956	37.591	9.78	0.00462
**R**	5	0.212	0.0425	-	-	5	0.135	0.0271	-	-	7	26.906	3.844	-	-
**LF**	3	0.203	0.0678	14.6	0.0646	3	0.122	0.409	6.36	0.1388	3	0.276	0.092	4.79	0.08193
**PE**	2	0.0093	0.0046	-	-	2	0.013	0.0064	-	-	4	0.076	0.019	-	-

**Factor**	**Coefficient**			***p*-value**		**Coefficient**			***p*-value**		**Coefficient**			***p*-value**	

Intercept	−1009.78			0.000010		−283.64			0.000230		−675.53			0.000000	
pH	225.63			0.000011		−63.54			0.000246		42.79			0.000002	
pH^2^	−21.11			0.000009		−7.36			0.000098		−3.48			0.000000	
T	15.28			0.000010		5.20			0.000163		18.32			0.000000	
T^2^	−0.11			0.000006		−0.04			0.000049		−0.13			0.000000	
pH.T	−0.26			0.000672		0.11			0.009356		−0.096			0.002283	

Notes: β-glucosidase: R^2^ = 0.99; *F* listed 5% = 5.05; β-xylosidase: R^2^ = 0.99; *F* listed 5% = 5.05; Xylanase: R^2^ = 0.98; *F* listed 5% = 3.97; M = Model; R = residual; LF = Lack of fit; PE = Pure error; df: degrees of freedom; SS: sum of squares; MS: mean square.
